# Improving Preoperative Care in Pakistan: An Evaluation of Pre-anesthetic Record Completeness and Documentation Practices

**DOI:** 10.7759/cureus.79761

**Published:** 2025-02-27

**Authors:** Asfa Mumtaz, Rimsha Zahid, Raza Sherazi, Nabiha Aslam, Kainaat Shakoor, Waleed Bin Waris, Zara Sohail, Muhammad Zuama Zafar Butt, Muhammad Bilal Ahmad, Farrukh Ansar

**Affiliations:** 1 Department of Surgery, Quaid-e-Azam International Hospital, Islamabad, PAK; 2 Department of Medicine, Quaid-e-Azam International Hospital, Islamabad, PAK; 3 Department of Emergency Medicine, Quaid-e-Azam International Hospital, Islamabad, PAK; 4 Department of Anesthesiology, Quaid-e-Azam International Hospital, Islamabad, PAK; 5 Department of Medicine, Alkhidmat Raazi Hospital, Rawalpindi, PAK

**Keywords:** documentation quality, healthcare quality, patient safety, pre-anesthetic assessment, surgical outcomes

## Abstract

Introduction

Accurate pre-anesthetic assessments are crucial for safe and effective anesthesia management. However, the completeness and quality of these assessments are often suboptimal, potentially impacting patient safety and surgical outcomes. This study evaluates the quality of preoperative assessments documented by anesthetists at a large private tertiary care hospital in Islamabad, Pakistan, focusing on adherence to standardized protocols and identifying specific deficiencies in documentation.

Methods

A retrospective descriptive study was conducted, reviewing 122 patient records from the General Surgery Department between October and December 2024. Pre-anesthetic record (PAR) forms were evaluated using a custom data collection tool based on the Global Quality Index (GQI). The tool assessed 16 key criteria for completeness, with each criterion categorized as "Yes-Complete" (fully documented with sufficient detail), "Yes-Incomplete" (partially documented but lacking essential details for comprehensive preoperative evaluation), or "No" (entirely missing). Statistical analysis was performed using descriptive statistics and IBM SPSS Statistics for Windows, V. 26.0 (IBM Corp., Armonk, NY, USA).

Results

The study found significant variability in the completeness of documentation. Patient demographics (age and name) were consistently recorded in 122 (100%) of the cases. However, critical data such as patient weight was recorded in only three (2.5%) of the forms, with 119 (97.5%) missing this information. Preoperative diagnoses were documented in one (0.8%) case, while 121 (99.2%) forms lacked this data. Preoperative vital signs were recorded in one (0.8%) case, with 120 (98.4%) missing them. Pre-medication prescriptions were noted in only two (1.6%) cases, leaving 120 (98.4%) incomplete.

Conclusion

The findings highlight substantial gaps in pre-anesthetic documentation. There is a pressing need for standardizing documentation practices to improve the quality and completeness of preoperative assessments.

## Introduction

Preoperative assessment is a critical component of perioperative care, providing a structured framework to evaluate and mitigate risks associated with anesthesia and surgery [1]. The quality of preoperative documentation, particularly in resource-limited healthcare settings, has a significant impact on patient safety and surgical outcomes [1]. Effective pre-anesthetic evaluations facilitate the identification of potential complications, optimize patient management, and improve perioperative care delivery [2]. However, in many healthcare systems, inconsistencies in the completeness and quality of pre-anesthetic records (PARs), which document a patient's medical history, physical examination findings, anesthesia risk assessment, and planned anesthetic management, persist due to factors such as inadequate staff training, lack of standardized documentation tools, and time constraints, often resulting in suboptimal patient outcomes. Standardized documentation refers to structured, protocol-based recording of preoperative evaluations, ensuring that all critical elements necessary for safe anesthesia care are consistently included [3]. These challenges underscore the importance of evaluating and improving pre-anesthetic documentation practices.

Pakistan's healthcare system faces numerous challenges related to healthcare delivery, including variability in clinical documentation standards [4]. The lack of standardized protocols for preoperative assessments, coupled with the resource constraints of tertiary care hospitals, exacerbates these challenges [4]. Despite the pivotal role of pre-anesthetic evaluations in ensuring safe surgical care, limited research has been conducted in Pakistan to assess the quality of PARs systematically. This paucity of data highlights the need for robust evaluations to identify deficiencies and establish benchmarks for improvement [5].

Global frameworks, such as the Global Quality Index (GQI) developed by Ausset et al., have provided valuable tools for assessing the quality and completeness of pre-anesthetic documentation [6]. These criteria emphasize the importance of recording demographic information, medical history, clinical findings, and risk assessments comprehensively [6]. Utilizing such tools in the context of Pakistan can help evaluate existing practices and identify areas requiring intervention. In this study, the original GQI criteria were applied without modification, as they align well with standard pre-anesthetic documentation practices in our setting.

This study was conducted at a large private tertiary care hospital in Islamabad, Pakistan, to evaluate the quality of pre-anesthetic documentation by anesthetists. The primary objective of this study is to evaluate the quality of pre-anesthetic documentation by analyzing key components such as demographic details, medical history, clinical findings, and risk classifications. By systematically reviewing PARs for patients undergoing surgery, this research aims to identify gaps in documentation practices, assess compliance with standardized criteria, and provide insights into areas needing improvement. The study's retrospective descriptive design allows for a comprehensive analysis of existing practices, enabling the identification of patterns and deficiencies that can inform future interventions.

The significance of this study lies in its potential to enhance preoperative care standards and improve patient safety in Pakistan while also contributing to the global effort to standardize and improve anesthesia documentation. By analyzing 16 predefined criteria adapted from the GQI framework [6], this study evaluates key components of pre-anesthetic documentation, including demographic details, medical history, clinical findings, and risk classifications. Addressing gaps in documentation, such as incomplete recording of preoperative vital signs and medication details, is crucial not only for improving perioperative care in resource-limited settings but also for enhancing anesthesia safety worldwide. Standardized protocols and continuous professional development for anesthetists can help mitigate risks associated with incomplete preoperative assessments. Additionally, the findings provide a foundation for future research exploring how improved documentation practices can lead to better patient outcomes, contributing to global anesthesia safety initiatives.

## Materials and methods

This was a retrospective descriptive study conducted at Quaid-e-Azam International Hospital in Islamabad, Pakistan, to evaluate the quality of preoperative assessments documented by anesthetists. A retrospective design was chosen as it allows for an objective evaluation of existing documentation practices without influencing real-time clinical decision-making. During the study period from October 2024 to December 2024, a total of 122 patients were scheduled for surgery. A surgical logbook containing a record of all patients scheduled for elective and emergency surgeries was systematically reviewed to identify a representative sample.

All surgical cases from the General Surgery Department between October 2024 and December 2024 were included in the study. A total of 122 patient files were retrieved from the medical records department. No exclusion criteria were applied, as the study aimed to evaluate the completeness and quality of pre-anesthetic documentation across all cases. This comprehensive inclusion ensured that the study reflected the full range of surgeries conducted during this period, minimizing selection bias and providing a complete dataset for analysis.

Data collection

Patient files included documentation such as clinical notes and pre-anesthetic evaluation records typically completed by anesthetists. A data collection form (see Appendices) was designed by the researcher based on criteria adapted from GQI by Ausset et al. [6]. Table 1 shows a predefined criteria used to evaluate the completeness of the selected PAR forms. The form included 16 key criteria, phrased as questions with standardized response options of "Yes-Complete", "Yes-Incomplete", or "No". The terms "Yes-Complete", "Yes-Incomplete", or "No" were used to evaluate the completeness of information documented in patient files. "Yes-Complete" indicated that all required details for a specific criterion were fully documented, while "Yes-Incomplete" meant that some information was recorded but lacked key elements. "No" was assigned when no documentation was present for the criterion. This system provided a structured method to assess the thoroughness and quality of preoperative assessments. Each question was formulated to assess critical components of preoperative evaluation, reflecting practices that influence patient outcomes. Data extraction was conducted by two independent groups, each consisting of three researchers, to ensure accuracy and consistency. Both groups independently reviewed the records, and their findings were subsequently cross-checked. Prior to data collection, all researchers underwent training to standardize the abstraction process and minimize variability. Any discrepancies between the groups were resolved through consensus discussions, with a senior investigator reviewing and approving the final dataset. This systematic approach enhanced reliability and reproducibility in identifying and evaluating pre-anesthetic documentation. 

**Table 1:** Predefined criteria used to evaluate the completeness of the selected PAR forms Yes-Complete: the documentation fully meets the specified criterion, with all required details accurately recorded. Yes-Incomplete: the documentation partially meets the specified criterion, with some relevant information recorded but missing key details necessary for completeness*. *No: the documentation does not meet the specified criterion, with no relevant information recorded. ASA: American Society of Anesthesiologists; PAR: pre-anesthetic record

Category	For outcome: Yes-Complete	For outcome: Yes-Incomplete	For outcome: No
Overall	PAR form present in the patient file and all 16 subsequent questions completed in accordance with the criteria used	PAR form present in the patient file but one or more of the 16 subsequent questions not completed in accordance with the criteria used	No PAR form in the patient file
General			
Patient's age	Patient's age provided	Patient's age provided but illegible	Field blank
Patient's name	Patient's name provided	Patient's name provided but illegible	Field blank
Patient's weight	Patient's weight recorded	Patient's weight recorded but illegible	Field blank
Medical history			
Allergies	Allergies recorded or "no allergies" specified	Allergies recorded with uncertainty (question mark noted)	Field blank
Anesthetic history and complications	Anesthetic history complication or "negative" was checked as required	Previous surgery was recorded but no anesthetic history made known	Field blank or no check mark
Previous surgeries	Previous surgeries recorded and dates specified	Previous surgeries recorded but dates not specified	Field blank
Preoperative evaluation			
Surgical procedure	Section for "proposed operation" completed	Surgical procedure provided but illegible	Field blank
Current medications	Medication(s) provided and dosage specified	Medication(s) provided but without specifying dosage	Field blank
Pre-medication prescribed	Pre-medication prescribed and dosage specified	Pre-medication prescribed but without specifying the dosage	No pre-medication prescribed
Preoperative diagnosis	Preoperative diagnosis recorded	Preoperative diagnosis recorded with uncertainty (question mark noted)	No preoperative diagnosis recorded
Preoperative vital signs	Respiration rate, pulse, and blood pressure recorded	One or two, but not all, preoperative vital signs recorded	No preoperative vital signs recorded
Clinical examination findings	Cardiovascular, respiratory, and neurological examination findings recorded	One or two, but not all, clinical findings recorded	No clinical examination findings recorded
Airway assessment	Neck and Mallampati assessment completed	Either neck or Mallampati assessment completed but not both	Field blank
"Per os" status	Last oral intake recorded in the section "Immediate pre-op assessment"	Last oral intake recorded with uncertainty (question mark noted)	Field blank
ASA status	ASA risk classification recorded	Other risk classifications recorded	Field blank
Special investigation results	Special investigations relevant to diagnosis and proposed surgery requested/done and recorded	One or more, but not all, special investigations relevant to diagnosis and proposed surgery requested/done and recorded	Field blank

To ensure consistent evaluation, predefined measures and criteria were applied to determine the completeness and accuracy of each parameter. The validity of the criteria was supported by their relevance to the most common complications associated with anesthesia as identified in the literature.

Pilot study

A pilot study was conducted using 15 randomly selected forms to evaluate the clarity and practicality of the data collection tool. These forms were reviewed by two consultant anesthesiologists for feedback and approval. Based on their recommendations, minor adjustments were made to improve the form's accuracy and usability. The pilot data were included in the final analysis as they met the eligibility criteria.

Data analysis

The finalized data from the reviewed forms were entered into a Microsoft Excel spreadsheet (Microsoft Corp., Redmond, WA, USA) for initial organization and preprocessing. Once completed, the dataset was imported into IBM SPSS Statistics for Windows, V. 26.0 (IBM Corp., Armonk, NY, USA), for detailed statistical analysis. Descriptive statistics, including frequencies and percentages, were used to summarize the completeness of documentation for each criterion. Inferential statistical tests were applied where appropriate to identify patterns or significant findings within the data. The collected data were analyzed descriptively, with results presented as frequencies and percentages. Comparisons were made between complete, incomplete, and missing entries for each criterion.

## Results

The evaluation of the completeness of PAR forms revealed significant variability across different sections. Below is a detailed discussion of each variable. Table 2 shows detailed results of the completeness of PAR.

**Table 2:** Completeness of pre-anesthetic record variables ASA: American Society of Anesthesiologists

Variable	Complete (n, %)	Incomplete (n, %)	Missing (n, %)
Patient's age	122 (100.0%)	0 (0.0%)	0 (0.0%)
Patient's name	122 (100.0%)	0 (0.0%)	0 (0.0%)
Patient's weight	3 (2.5%)	0 (0.0%)	119 (97.5%)
Allergies	120 (98.4%)	0 (0.0%)	2 (1.6%)
Anesthetic history and complications	115 (94.3%)	0 (0.0%)	7 (5.7%)
Previous surgeries	107 (87.7%)	0 (0.0%)	15 (12.3%)
Surgical procedure	117 (95.9%)	0 (0.0%)	5 (4.1%)
Current medications	117 (95.9%)	0 (0.0%)	5 (4.1%)
Pre-medication prescribed	2 (1.6%)	0 (0.0%)	120 (98.4%)
Preoperative diagnosis	1 (0.8%)	0 (0.0%)	121 (99.2%)
Preoperative vital signs	1 (0.8%)	1 (0.8%)	120 (98.4%)
Clinical examination findings	118 (96.7%)	0 (0.0%)	4 (3.3%)
Airway assessment	103 (84.4%)	2 (1.6%)	17 (13.9%)
"Per os" status	99 (81.1%)	0 (0.0%)	23 (18.9%)
ASA status	92 (75.4%)	0 (0.0%)	30 (24.6%)
Special investigation results	92 (75.4%)	9 (7.4%)	21 (17.2%)

General information

The recording of patient demographics showed mixed results in terms of completeness. The patient's age was documented in 100% of cases (N = 122), demonstrating a high standard of compliance for this critical demographic variable. Similarly, the patient's name was recorded accurately and without any issues in all cases (N = 122), ensuring reliable traceability and identification. However, significant gaps were observed in the documentation of the patient's weight, with only 2.5% of forms (N = 3) including this essential parameter, leaving 97.5% (N = 119) incomplete.

Medical history

Allergy information was documented in nearly all cases, with 98.4% of forms (N = 120) accurately recording the presence or absence of allergies. Only 1.6% of forms (N = 2) were missing this important information, reflecting a high level of compliance. Similarly, anesthetic history and complications were recorded in 94.3% of forms (N = 115), leaving 5.7% (N = 7) incomplete. Documentation of previous surgeries was slightly less thorough, with 87.7% of cases (N = 107) including relevant details, while 12.3% of forms (N = 15) omitted this data.

Preoperative evaluation

The documentation of the surgical procedure showed high compliance, with 95.9% of forms (N = 117) containing the proposed operation details. However, 4.1% of forms (N = 5) lacked this information. Similarly, current medications were recorded in 95.9% of cases (N = 117), leaving a small but significant gap of 4.1% (N = 5). In contrast, the documentation of pre-medication prescriptions was notably poor, with only 1.6% of forms (N = 2) containing complete information. A significant 98.4% of forms (N = 120) failed to document this aspect.

The recording of preoperative diagnoses was alarmingly low, with only 0.8% of forms (N = 1) providing this information and 98.4% (N = 120) entirely missing it, representing a major shortfall in clinical documentation. Preoperative vital signs were equally underreported, with complete records in just 0.8% of cases (N = 1) and partial documentation in another 0.8% (N = 1). A striking 98.4% of forms (N = 120) lacked any record of preoperative vital signs, emphasizing an urgent need to address this deficiency in documentation practices.

Clinical findings and assessment

Clinical examination findings were comprehensively documented in 96.7% of cases (N = 118), with only 3.3% (N = 4) remaining incomplete, highlighting this as a strong area of compliance. Airway assessments, although complete in 84.4% of cases (N = 103), were incomplete in 1.6% (N = 2) and entirely missing in 13.9% (N = 17). Similarly, the last oral intake, a critical parameter for safe anesthesia, was documented in 81.1% of cases (N = 99), leaving a notable 18.9% (N = 23) without this information.

Risk classification and investigations

The American Society of Anesthesiologists (ASA) classification was documented in 75.4% of cases (N = 92), while the remaining 24.6% (N = 30) lacked this information. Similarly, the recording of special investigation results showed variability, with 75.4% of cases (N = 92) documented fully, 7.4% (N = 9) partially completed, and 17.2% (N = 21) entirely missing.

## Discussion

Preoperative anesthesia assessment is an essential evaluation before surgery, aimed at detecting potential risks and ensuring safe management during and after the procedure [7]. This process involves the assessment and optimization of medical treatment, a detailed discussion of the anesthesia plan with the patient, and obtaining informed consent [7]. The goal is to anticipate potential complications and implement preventive measures, forming the cornerstone of preoperative medical management [8]. This audit was conducted at a tertiary care hospital, reviewing the data of 122 patients, including both elective and emergency cases. Significant variability was observed in the completeness of PAR forms in terms of adherence to criteria.

The standardized PAR forms were available in 100% (N = 122) of the patient medical records, which surpasses the findings of an audit by Mokgwathi et al., where only 85.2% of patient records contained the necessary documentation [9]. This higher rate of compliance highlights the robust system in place at this hospital for maintaining accurate medical records. The completeness of patient identification information (name and age) was flawless, with 100% (N = 122) of cases documenting these parameters accurately. This adherence to documentation is significantly better than that observed in an audit by Swart and Kuhn, where patient identification was documented correctly in 92.6% of cases [10].

However, a notable gap was identified in the documentation of patient weight, recorded in only 2.5% (N = 3) of the forms. Weight is crucial for the accurate calculation of anesthetic drug dosages, influencing both the effectiveness and safety of the anesthesia plan [11]. This stark contrast with audits by Mokgwathi et al. (65.3%) and Swart and Kuhn (75.3%) suggests a potential oversight in ensuring this essential data is systematically recorded [9,10]. Given the significant role of weight in dosing and anesthetic timing, steps should be taken to improve adherence to this documentation practice. Regular training for medical staff and reinforcing the importance of this data might improve compliance [12]. Practical interventions could include implementing a standardized checklist or integrating automated prompts into the patient intake system to remind clinicians to record weight.

Food and drug allergies were documented in 98.4% (N = 120) of patient records, which is a positive finding. This information is vital in preventing potentially life-threatening perioperative reactions, such as anaphylaxis [13]. A strong preoperative allergy history can guide the anesthesiologist in avoiding triggers and ensuring the patient receives appropriate prophylaxis [13]. Furthermore, the anesthetic history, including complications from previous anesthesia episodes, was documented in 94.3% (N = 115) of cases. Such documentation is crucial because it allows the anesthesia team to anticipate and manage potential complications, thereby reducing the risk of perioperative incidents [14].

The documentation of patients' previous surgeries was noted in 87.7% (N = 122) of the cases. Although this is relatively high, the gap in this information could lead to suboptimal anesthesia planning and inadequate consideration of postoperative complications [15]. Previous surgeries, particularly major ones, may alter the patient's anatomy or response to anesthesia, necessitating special preparation [15]. Therefore, the importance of recording this information in every case cannot be overstated.

The documentation of the surgical procedure to be performed and the patient's current medication was found to be accurate in 95.9% (N = 117) of the cases. This is critical as understanding the nature of the surgery allows the anesthesia team to tailor their plan accordingly, ensuring the proper administration of anesthetics and other intraoperative medications. One of the most concerning findings was the very low documentation of pre-medication prescriptions, recorded in only 1.6% (N = 2) of the cases. Pre-medications, such as anxiolytics or antiemetics, play an essential role in preparing the patient for anesthesia and preventing postoperative complications like nausea or anxiety [16]. The absence of proper documentation in this area presents a clear patient safety risk, potentially leading to delays in recovery or increased discomfort for the patient [16]. It is crucial to address this gap by ensuring pre-medication prescriptions are clearly documented and reviewed during preoperative assessments.

The documentation of preoperative diagnosis and vital signs was alarmingly low at 0.8% (N = 1). Vital signs, such as blood pressure, heart rate, and respiratory rate, are fundamental for assessing the patient's overall health status and readiness for surgery [17]. The absence of this important information compromises the ability of the anesthesia team to make necessary adjustments and prepare for any changes that might occur during surgery. Vital signs also help detect potential perioperative complications, such as arrhythmias or changes in blood pressure, which can affect anesthesia management [18].

Clinical examination findings were documented in 96.7% (N = 118) of the cases, reflecting strong adherence to this aspect of the preoperative evaluation [3]. A thorough clinical examination is essential for identifying any underlying conditions or risks that could affect anesthesia and surgery [3]. It helps the anesthesia team prepare for potential complications, such as a compromised airway or cardiovascular instability [3].

Airway assessment, an essential component of the preoperative evaluation to ensure safe intubation and ventilation during surgery, was documented in 84.4% (N = 103) of cases, with partial completion in 1.6% (N = 2). While this is a relatively high percentage, airway management remains one of the most critical aspects of anesthesia [19]. Incomplete or insufficient airway assessment can lead to severe complications, including difficulty in securing the airway during induction, which can endanger the patient's life [20]. This area requires further attention, particularly through improved training and better adherence to guidelines.

ASA status was recorded in 75.4% (N = 92) of the cases, a lower figure than expected. The ASA classification is a well-established tool for assessing preoperative risk and predicting perioperative outcomes based on the patient's medical history and physical condition [21]. This lower documentation rate suggests that ASA status may not be consistently utilized as part of the preoperative evaluation, which could compromise the ability to predict and mitigate perioperative risks. Ensuring consistent documentation of ASA status should be emphasized in training and quality assurance initiatives.

Results of special investigations, such as laboratory tests or imaging studies, were documented in 75.4% (N = 92) of the cases, with partial completion noted in 7.4% (N = 9). For patients with comorbidities or higher perioperative risks, such as those with cardiovascular or renal disease, special investigations are critical for tailoring anesthesia plans and ensuring patient safety [22]. Inadequate or incomplete documentation of these investigations could result in undiagnosed issues or inadequate preparation.

The findings from this clinical audit underscore critical gaps in preoperative anesthesia assessments. Inadequate documentation of variables such as weight, preoperative vital signs, airway assessments, and ASA status can compromise the anesthesia team's ability to develop a personalized and safe anesthesia plan. These shortcomings highlight the need for targeted interventions, including regular training for clinicians, more stringent documentation protocols, and periodic audits to provide feedback and improve compliance. Ensuring that all critical parameters are comprehensively documented will significantly enhance patient safety and contribute to better perioperative outcomes.

This study offers valuable insights into the quality of preoperative assessments documented by anesthetists. Its comprehensive inclusion of 122 patient files ensures a representative sample and minimizes selection bias. The use of predefined criteria adapted from the GQI enhances the consistency and rigor of the evaluation, providing a structured method to assess the thoroughness of documentation. However, the study's retrospective design limits its ability to establish causal relationships and may introduce information bias, as incomplete or missing records may not fully reflect actual clinical practices. Additionally, the research was conducted in a single private tertiary care hospital in Islamabad, which restricts the generalizability of the findings to other healthcare settings or regions. The observed variability in the completeness of documentation, particularly in critical areas such as preoperative diagnoses and vital signs, suggests potential gaps in clinician training or system-level inefficiencies, though the study does not explore these underlying factors. These limitations, coupled with the lack of real-time data on corrective measures, highlight the need for further research to explore the root causes of documentation deficiencies and evaluate interventions aimed at improving preoperative assessment quality.

## Conclusions

The results of this clinical audit underscore significant deficiencies in the completeness of the PAR forms, highlighting critical documentation gaps that could compromise patient safety. While areas like patient identification, allergies, and anesthetic history showed commendable compliance, essential parameters such as preoperative vital signs, patient weight, ASA status, pre-medication prescriptions, and preoperative diagnoses were notably underreported. These omissions can hinder the anesthesia team's ability to develop safe, individualized care plans, potentially increasing the risk of perioperative complications. The findings call for immediate action, with a focus on prioritizing the documentation of vital signs and ASA status, given their direct impact on anesthesia planning. Targeted interventions such as comprehensive clinician training, standardized documentation protocols, and regular audits are essential to enhance documentation practices. Addressing these gaps is crucial to improving perioperative care, ensuring accurate risk assessment, and ultimately promoting better patient outcomes.

## Appendices

**Table 3:** Questionnaire/data sheet to collect data Pre-op anesthesia evaluation of surgery patients: predefined criteria used to evaluate the completeness of the selected PAR forms ASA: American Society of Anesthesiologists; PAR: pre-anesthetic record

Category	For outcomes: Yes-Complete	For outcomes: Yes-Incomplete	For outcomes: No
Overall			
PAR form			
General			
Patient's age			
Patient's name			
Patient's weight			
Medical history			
Allergies			
Anesthetic history and complications			
Previous surgeries			
Preoperative evaluation			
Surgical procedure			
Current medications			
Pre-medication prescribed			
Preoperative diagnosis			
Preoperative vital signs			
Clinical examination findings			
Airway assessment			
"Per os" status			
ASA status			
Special investigation results			
